# Why double-stranded RNA resists condensation

**DOI:** 10.1093/nar/gku756

**Published:** 2014-08-14

**Authors:** Igor S. Tolokh, Suzette A. Pabit, Andrea M. Katz, Yujie Chen, Aleksander Drozdetski, Nathan Baker, Lois Pollack, Alexey V. Onufriev

**Affiliations:** 1 Department of Computer Science, Virginia Tech, Blacksburg, VA 24061, USA; 2 School of Applied and Engineering Physics, Cornell University, Ithaca, NY 14853-3501, USA; 3 Department of Physics, Virginia Tech, Blacksburg, VA 24061, USA; 4 Applied Statistics and Computational Modeling Group, Pacific Northwest National Laboratory, Richland, WA 99352, USA

## Abstract

The addition of small amounts of multivalent cations to solutions containing double-stranded DNA leads to inter-DNA attraction and eventual condensation. Surprisingly, the condensation is suppressed in double-stranded RNA, which carries the same negative charge as DNA, but assumes a different double helical form. Here, we combine experiment and atomistic simulations to propose a mechanism that explains the variations in condensation of short (25 base-pairs) nucleic acid (NA) duplexes, from B-like form of homopolymeric DNA, to mixed sequence DNA, to DNA:RNA hybrid, to A-like RNA. Circular dichroism measurements suggest that duplex helical geometry is not the fundamental property that ultimately determines the observed differences in condensation. Instead, these differences are governed by the spatial variation of cobalt hexammine (CoHex) binding to NA. There are two major NA-CoHex binding modes—internal and external—distinguished by the proximity of bound CoHex to the helical axis. We find a significant difference, up to 5-fold, in the fraction of ions bound to the external surfaces of the different NA constructs studied. NA condensation propensity is determined by the fraction of CoHex ions in the external binding mode.

## INTRODUCTION

Highly charged DNA molecules are expected to repel each other, yet can be condensed by certain multivalent ions into structured aggregates (1–3). The condensation phenomenon is biologically important. Compaction of anionic DNA and RNA molecules by oppositely charged cationic agents enables efficient packaging of genetic material inside living cells and viruses (4–7). *In vitro* experiments on DNA in aqueous solution revealed that the cation-induced condensation requires the ion valence to be +3 or higher (1,8). Trivalent cations, such as cobalt(III) hexammine (CoHex) or spermidine, can effectively condense DNA while divalent inorganic cations (e.g. Mg^2+^) alone cannot, suggesting the significant contribution of electrostatic interactions to the counterintuitive effective attraction between nucleic acids (NA). Over the years, a number of experimental and theoretical studies have been carried out (9–28) with the goal of providing a general physical picture of the condensation phenomenon. A commonly accepted view has emerged: the effective attraction is mainly due to electrostatic contributions. However, the general picture as well as the atomistic mechanism of NA condensation, including the role of hydration forces (12,29) or multivalent counterion correlations (14,26), are still incomplete and cannot be fully explained.

Particularly puzzling are our recent experimental results which show that double-stranded (ds) RNA helices resist condensation under conditions where short DNA duplexes condense readily (30). The results are somewhat paradoxical since the multivalent counterions—whose attractive interactions with the oppositely charged duplex are key to the condensation—are expected (31) to bind more strongly to RNA than to DNA. The striking difference between DNA and RNA condensation has sparked renewed efforts to understand the molecular mechanism of NA condensation (32) beyond existing models (13,33–35) and phenomenological theories (27) based on simplified NA geometries.

Because dsDNA is typically found in B-type helical forms, while dsRNA stays in A-like forms, it was suggested that counterion distributions around the differing helical forms may explain the difference in condensation behavior of DNA and RNA (30). However, atomically detailed CoHex distributions are hard to measure in solution samples. In principle, all-atom explicit solvent molecular dynamics (MD) simulations (36–43) can provide the necessary details of NA interaction with multivalent ions, including ion distributions around NA, as well as preferred ion binding sites and their occupancies. To ensure equilibration of multivalent ion distributions, especially around RNA, such simulations will have to go well beyond the time scale of tens of nanoseconds—the longest reported atomistic MD simulation to date of small DNA fragments interacting with tri- or tetravalent ions in solution (39).

To uncover the mechanism of RNA resistance to condensation and to determine key factors responsible for different NA condensation propensities, we studied CoHex-induced condensation and CoHex binding properties of four short NA helical constructs using experiments and experimentally guided atomistic MD simulations on appropriate time scales.

## MATERIALS AND METHODS

### Materials

Single-stranded NA were purchased from IDT. Four ds NA constructs were made by annealing in STE buffer (50 mM NaCl, 10 mM TRIS, 1 mM ethylenediaminetetraacetic acid, pH 7.4) at 94°C for 2 min. The 25 bp duplex DNA, RNA and DNA:RNA hybrid all used the same mixed sequence (GCA TCT GGG CTA TAA AAG GGC GTC G, with T replaced by U in RNA strands) employed in our previous studies (30,31,44). For the homopolymeric 25 bp poly(dA):poly(dT) DNA duplex, an ultraviolet (UV) melt was taken to verify monophasic annealing. CoHex chloride powder was purchased from Sigma-Aldrich and dissolved in water. CoHex concentrations were verified by measuring the absorption at 473 nm using extinction coefficient 56.2 M^−1^cm^−1^ as reported by Deng and Bloomfield (45). Duplexes were dialyzed in pH 7.0 Na-MOPS buffer containing NaCl.

### CoHex-induced NA condensation as monitored by UV absorption

**Figure 1.**
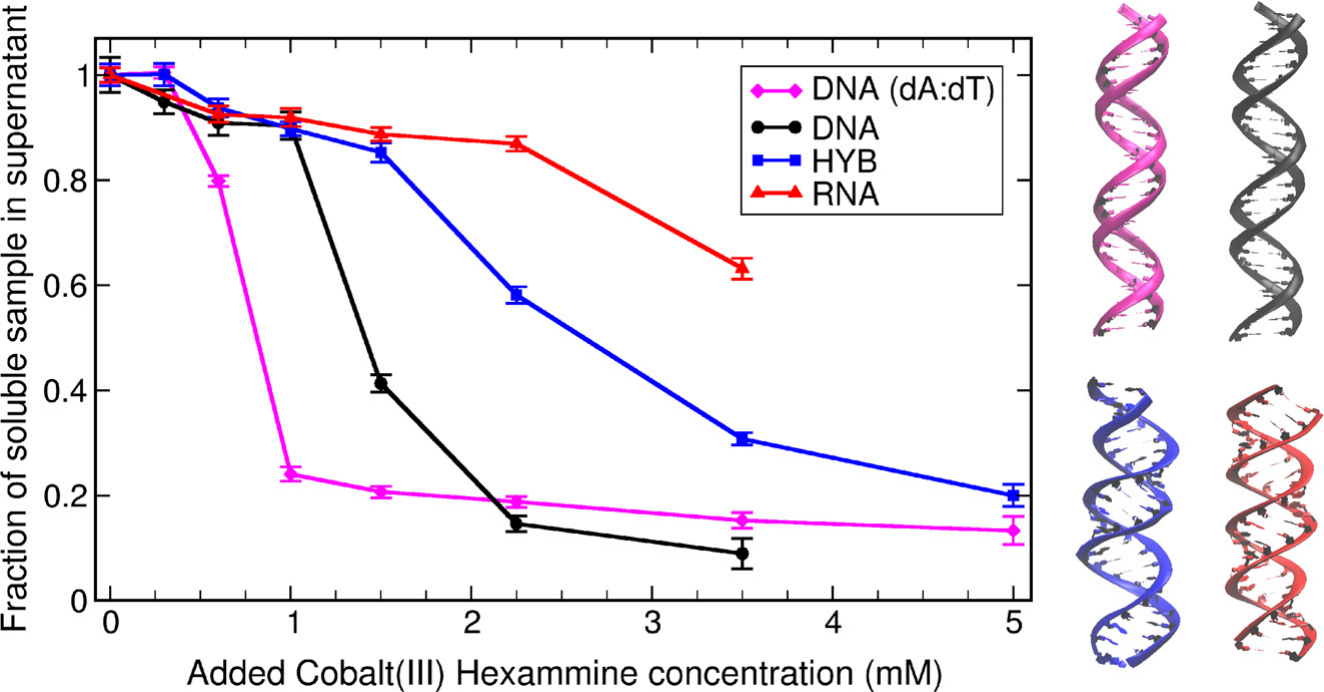
Fraction of unprecipitated short 25 bp NA duplexes (DNA homopolymer, mixed sequence DNA, RNA and DNA:RNA hybrid) calculated from UV absorption as a function of CoHex concentration in solution (starting duplex concentration is 40 μM). The condensation propensity of each duplex is characterized by the CoHex concentration at the midpoint of duplex condensation—the higher the value the lower the propensity. The right panel illustrates the overall suggested structures of these duplexes as either B-form or A-like form helices. The color matches the lines in the panel on the left.

The NA constructs were dialyzed in 20 mM NaCl and 1 mM pH 7.0 Na-MOPS buffer and separated in 40 μM 100 μl aliquots. Each aliquot was spiked with 5 μl of concentrated CoHex and incubated at 4°C for 2 h. The aliquots were centrifuged at 10 000 revolutions per minute for 10 min and the supernatant was separated for absorption spectroscopy measurements. The absorption spectra were recorded from 220 to 600 nm using a Cary 50 spectrophotometer. The optical density at 260 nm (OD_260_) was monitored to determine the amount of NA left in the solution. The recorded value was normalized using the OD_260_ of the sample with no added CoHex and reported in Figure 1 as the fraction of soluble sample in the supernatant. The error bars shown were determined by propagation of errors due to baseline differences at OD_600_ and presence of residual CoHex at OD_473_.

### Circular dichroism (CD)

CD measurements were used to verify the helical form of the double helices and examine the effect of added 1 mM CoHex. The NA concentrations were 20 μM. Samples (20 μl) were kept at room temperature in a solution of 100 mM NaCl and 1 mM pH 7.0 Na-MOPS buffer. Since NA condensation is modulated by the amount of monovalent ions present in solution (8,24), we chose higher NaCl concentration to prevent the spontaneous condensation observed in Figure 1. These conditions complement the monovalent ion concentrations used for X-ray scattering experiments reported in (30). Measurements were made on a BioLogic MOS 450 configured in CD mode. Five scans per spectrum, each from 200–320 nm with 5 s/nm steps, were used for averaging.

### All-atom MD simulations

All MD simulations of the 25 bp DNA, RNA and hybrid duplexes were carried out using AMBER12 (46) and ff99bsc0 force field (47,48). The 25 bp B′-form structure of poly(dA·dT) duplex was constructed from the helical parameters of 1PLY (PDB) X-ray structure (49) using 3D-DART server (50) and 3DNA software (51). P-O3′ bonds were optimized by energy minimization keeping atoms P,OP1,OP2 and N* restrained (100 kcal/mol/Å^2^ force constant). The 25 bp mixed sequence DNA and RNA duplexes were constructed in canonical B- and A-form, respectively, using Nucleic Acid Builder (NAB) (52). The 25 bp mixed sequence DNA:RNA hybrid was constructed with the help of MD simulations. Two structures corresponding to canonical B- and A-forms were initially built using NAB-based ‘make-na’ server (http://structure.usc.edu/make-na/) by J. Stroud. Each structure was solvated with 16838 TIP3P water molecules, 72 Na^+^ and 24 Cl^−^ ions, and equilibrated, unrestrained, for 200 ns at 300 K. Both hybrid structures converged to essentially the same A-like structure. The final structure from the A-form trajectory was used for simulations of the DNA:RNA hybrid with CoHex. To approximate the experimental NA condensation conditions of a very low Na^+^ concentration and to avoid uncertainties associated with Na^+^ force field parameters (53), all of the following MD simulations, which involved CoHex, were carried out without any mobile ions other than CoHex. This approximation is justified because the experimental Na^+^ concentration is far lower than the concentrations necessary to suppress CoHex-induced DNA condensation at mM levels of CoHex (8) used here, and because a negligible effect of Na^+^ on the bound CoHex ions was observed at Na^+^ concentrations below 40 mM (11). For the simulations, each of four NA duplexes was solvated with 16880 TIP3P water molecules and 16 CoHex ions (neutralizing amount). After initial water minimization (2000 steps), the systems were equilibrated for 0.5 ns in canonical ensemble (NVT) and 0.5 ns in isothermal-isobaric ensemble (NPT) using 1 fs time step and achieving 1 atm pressure and 300 K temperature. The latter was maintained using Langevin dynamics with the collision frequency of 1 ps^−1^. Periodic boundary conditions and the particle mesh Ewald method were used. During the minimization and equilibration, all NA atoms were harmonically restrained with 100 kcal/mol/Å^2^ force constant. Then, using NVT ensemble and 2 fs time step, 320 ns production trajectories were generated for each system. For DNA atoms, the restrain force constant was reduced to 50 kcal/mol/Å^2^ in the production stage. The RNA and DNA:RNA hybrid duplexes were simulated unrestrained. The need for ∼300 ns trajectories was dictated by the long time scale fluctuations (50–100 ns) in the number of bound CoHex ions in the unrestrained systems (see Supplementary Data). CoHex distributions are presented as the average numbers of CoHex ions in consecutive thin (0.25 Å) cylindrical layers around duplexes. For these calculations, the first 40 ns of each trajectory were not taken into account. Robustness of the CoHex distributions to the choice of water model was confirmed, see Supplementary Data.

### Continuum electrostatics calculations

Electrostatic potentials and fields around the 25 bp mixed sequence B-DNA and A-RNA duplexes were calculated in continuum solvent approximation via the Poisson-Boltzmann (PB) equation with MEAD (54) 2.2.9 PB solver. Representative configurations of the duplexes with 15 bound CoHex ions were used. Grid spacing of 0.3 Å, dielectric constants of 4 (solute) and 80 (water), and solvent probe radius of 3 Å (CoHex radius) were employed. The potentials and fields were visualized using GEM software (55).

## RESULTS

### Experimental measurement of duplex condensation propensity

The propensity for condensation, which is accompanied by precipitation of NA duplexes, was probed by measuring the fraction of duplexes remaining in solution after the addition of CoHex. We examined 25 base-pair mixed-sequence duplexes of RNA and DNA, a mixed sequence DNA:RNA hybrid, as well as a DNA homopolymer made of poly(dA):poly(dT). As in Li *et al.* (30), this fraction was determined by measuring the change in UV absorption of the supernatant. Results, shown in Figure 1, reveal marked differences in the CoHex-induced condensation of the four different NA constructs.

A striking difference in condensation is measured in the RNA-containing structures (dsRNA and DNA:RNA hybrid) relative to the two DNA structures. Homopolymeric DNA begins to condense at the lowest added CoHex levels, followed by mixed-sequence DNA. The RNA-containing duplexes both require more added CoHex to precipitate. The DNA duplexes are suggested to be B-like, while the latter two are A-like (56–60). These results support our initial suggestion (30) that NA condensation propensities are related to NA duplex geometry. However, Figure 1 shows that this simplistic interpretation is not completely correct. Substantial differences in condensation are measured within each structural family. Of the two A-like structures, pure RNA requires more added CoHex to precipitate than does the DNA:RNA hybrid with the same sequence. Differences in B-type helices are detected as well: less added CoHex is required to condense homopolymeric poly(dA):poly(dT) than mixed-sequence DNA.

### NA duplexes that condense differently can have the same helical structure

To gain insight into the potential connection between duplex structure and condensation, we used CD spectroscopy to characterize each of the four NA duplexes. To account for changes induced by CoHex-NA binding, spectra were measured for each sample in the absence and presence of CoHex ions. CD provides unique experimental signatures of A- and B-form NA helices. In general, B-DNA has CD peaks at 220 and 275 nm and a dip at 245 nm (61), while A-form RNA has a peak at 260 nm and a dip at 210 nm (56).

**Figure 2.**
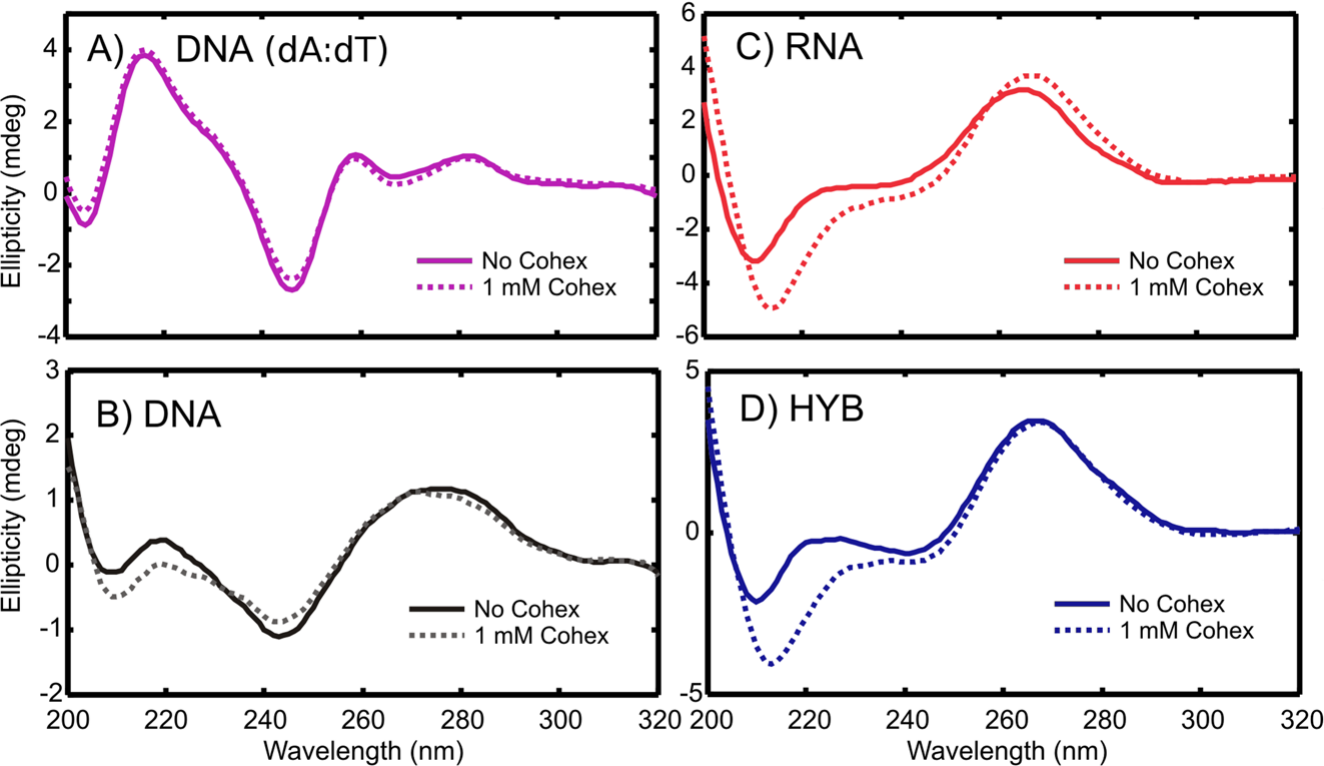
CD spectra of the NA helices with and without CoHex.

Figure 2 shows the CD spectra of our four NA duplexes. Not surprisingly, both DNA helices display B-like features. The spectrum of the homopolymeric poly(dA):poly(dT) duplex (Figure 2A) strongly resembles a B′-form (58) of DNA, with peaks at 217, 258 and 282 nm, and a dip at 247 nm (62). The mixed sequence DNA spectrum (Figure 2B) is typical of canonical B-form (63). No notable spectral shifts in the CD spectra of both DNA duplexes are observed after the addition of CoHex suggesting the helical forms of our 25 bp DNA structures are unaffected by the addition of CoHex. The homopolymer remains in B′-form and the mixed sequence DNA in the canonical B-form.

The spectra of the RNA and DNA:RNA hybrid duplexes without CoHex (Figure 2C and D) differ slightly from each other, but closely resemble A-form RNA (56). Upon addition of CoHex, both spectra slightly change. The differences between the CD spectra with and without CoHex are significant and above the experimental noise. With CoHex, the RNA and the hybrid become virtually identical. Therefore, since in the presence of CoHex both RNA containing duplexes are in practically the same A-like form, the observed differences in CoHex-induced condensation between DNA:RNA hybrid and RNA shown in Figure 1 cannot be explained solely based on differences in the helical structure parameters, as suggested in (32).

### Experiment guides atomistic modeling

The experimental CD spectra (Figure 2) indicate a substantial difference in the response of RNA and DNA structures to the addition of CoHex ions. The RNA and hybrid duplexes change their helical geometries while retaining the major features of A-form structure in the presence of CoHex. On the other hand, the structures of the DNA duplexes remain mostly unchanged. Thus, in our atomistic simulations we assume rigid B′- and B-form DNA structures, as enforced by applying positional restraints to all of the DNA atoms. This assumption of rigidity minimizes possible bias due to the use of imperfect modern force fields (64). In contrast, to reflect the measured CoHex-induced changes in both RNA and the hybrid structures, these molecules are simulated without any positional restraints. The MD simulations were performed to provide sufficient equilibration and sampling of bound configurations of 16 (neutralizing amount) CoHex ions in the simulation box, see ‘Materials and Methods’. After addition of CoHex, the simulations report that both the hybrid and the RNA structures relax to similar A-like form helices consistent with the CD experimental data.

### NA structure strongly determines differences in CoHex binding and distribution

**Figure 3.**
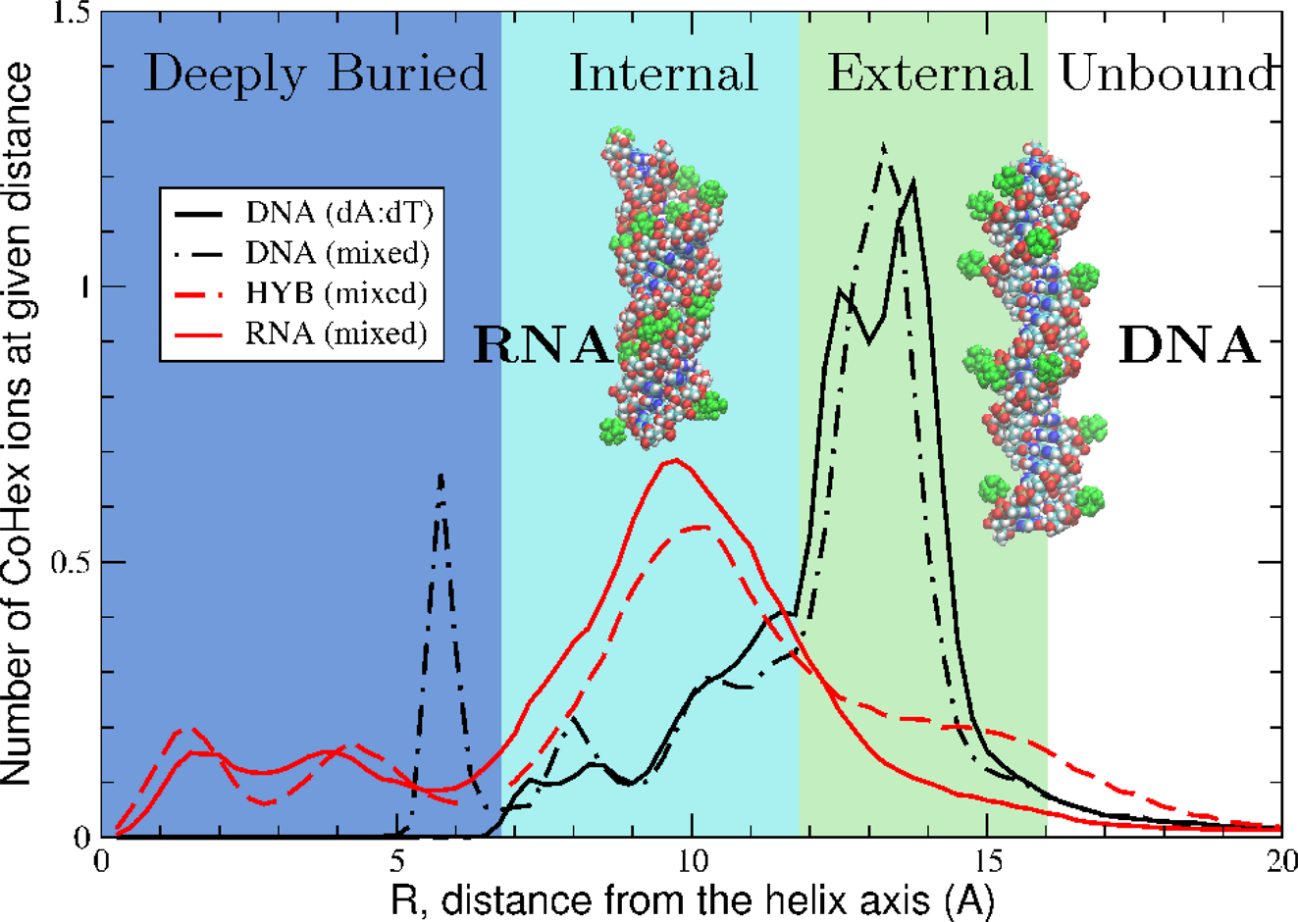
CoHex distributions and ion binding modes (shells) around four types of NA duplexes. DNA duplexes exhibit mostly external CoHex binding (12–16 Å). For RNA and DNA:RNA hybrid, most neutralizing CoHex ions are bound internally (7–12 Å). Shown are the average numbers of CoHex ions in thin (0.25 Å) cylindrical layers around duplexes, at the given distance *R* from the helical axis. Insets show representative snapshots of RNA (left) and DNA (right) structures with bound CoHex ions (green). In DNA, 80–100% of bound ions are localized at the surface of the phosphate backbone.

Sub-microsecond all-atom MD simulations (see ‘Materials and Methods’) reveal a detailed picture of CoHex distributions around the four 25 bp duplexes used in the condensation experiments, see Figure 3. In all four systems, CoHex ions bind preferentially to the phosphate groups whose centers are about 10 Å from the helical axis in all structures. However, the structural differences between the B- and A-form of NA, including differences in the width of the major grooves and in the relative orientation of the phosphate oxygens, lead to striking differences in CoHex binding, see Figure 3 and Supplementary Data. We identify two dominant spatial modes of CoHex binding: an *internal* mode for ions bound inside the major groove, 7–12 Å from the helical axis, and an *external* mode for ions bound at the external surface of the phosphate groups exposed to the bulk, 12–16 Å from the helical axis. As illustrated in the inset of Figure 3, the vast majority of CoHex ions in the *external* mode are outside the DNA major groove. The ions are mobile and mostly found ‘hovering over’ the phosphate backbone and the minor groove, see Supplementary Data for details. The cylindrical shells in the CoHex distributions, which correspond to these modes, are color-coded green and light blue in Figure 3. The upper boundary of the *external* binding shell roughly corresponds to the largest distance (from the axis) at which CoHex can still make direct contacts with the phosphate groups. Most CoHex ions are found within these two shells. A small fraction of ions are found deeply buried inside the major groove of DNA: about two CoHex ions are bound to the nucleotide bases at Guanine-phosphate-Cytosine (GpC) steps in DNA (65) and GpG steps in RNA and hybrid (66), see Supplementary Data. The existence of several CoHex binding modes in the CoHex-DNA interaction is in agreement with the heterogeneity in the magnetic environments sampled by CoHex (^59^Co NMR) adjacent to DNA (11,65).

For the RNA and hybrid, the simulations find most of the bound CoHex ions reside in the *internal* shell. These ions are buried within the major grooves with distribution peaks at 10 Å from the helical axis (see Figure 3 and Supplementary Data). In contrast, CoHex binds to both DNA structures by preferring the *external* binding mode, with the distribution peaks around 14 Å.

**Figure 4.**
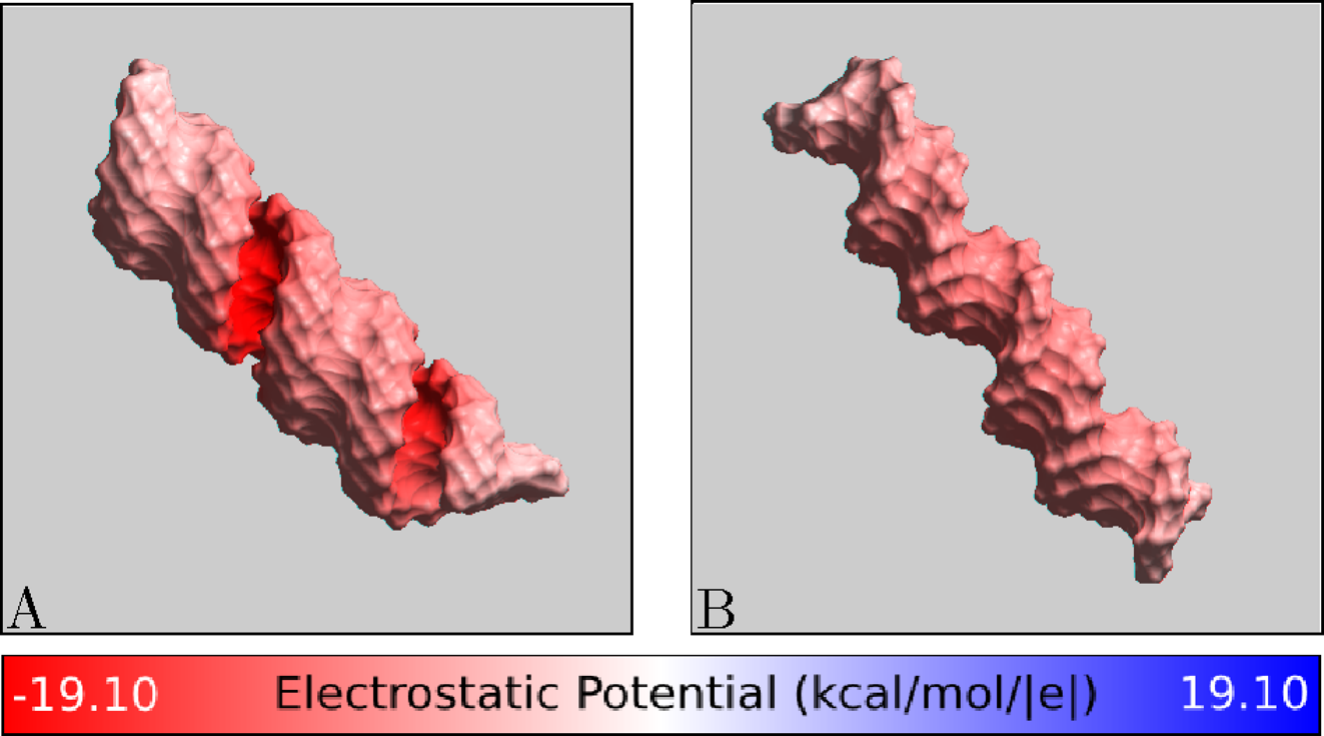
Electrostatic potential at the ‘CoHex-accessible’ surface of (**A**) A-form RNA and (**B**) B-form DNA structures, without bound CoHex ions. Shown is the electrostatic potential computed 3 Å away (CoHex radius) from the molecular surface. B-DNA minor groove is sterically inaccessible to large CoHex ions.

The major *external–internal* dichotomy in the preference for binding of CoHex counterions to B- and A-form NA is consistent with the major differences in the distribution of electrostatic potential around the NA surface (67) accessible to large CoHex ions, Figure 4. In the B-form geometry (DNA), the strength of the potential in the *internal* shell (major groove) is similar to that in the *external* shell. However, the *external* binding mode is energetically preferred: CoHex ions bound in the *external* shell are, on average, further apart and more exposed to solvent compared to internally bound CoHex, which are closer to the helical axis and therefore experience stronger ion–ion repulsion. In contrast, in the A-form geometry found in RNA and the hybrid structure, the negative electrostatic potential in the *internal* shell (major groove) is at least 10 kcal/mol/|*e*| stronger than in the *external* shell, see Figure 4. The resulting increase in the relative affinity of the *internal* shell to CoHex is sufficient to overwhelm the additional ion–ion repulsion, which explains why most of the CoHex ion bind internally in A-form NA, see Figure 3.

### The connection between the counterion distribution around NA and condensation

As CoHex counterions bind to free NA, the resulting charge neutralization patterns for A- and B-form duplexes are expected to be very different, consistent with the differences in the ion binding patterns. Since it is ultimately attraction between opposite charges that, under right conditions, leads to CoHex-induced NA condensation, we expect the differences in bound counterion distributions seen in Figure 3 to be directly relevant to the observed differences in the condensation behavior. Table 1 summarizes the results of our simulations and the experimental condensation propensities, Figure 1, and offers insight into the connection between counterion distribution and condensation. For each of the four duplexes, we list the number of CoHex ions in the *external*, *internal* and deeply buried binding shells; the degree of duplex neutralization afforded by the bound ions; and the relative condensation propensities determined from the experimental data shown in Figure 1.

**Table 1.** Relative measured condensation propensities from Figure 1 and average numbers of bound CoHex ions in each binding shell shown in Figure 3. 100% neutralization would correspond to 16 bound CoHex counterions

	DNA (dA:dT)	DNA	HYB	RNA
Condensation propensity	**highest**	**high**	**low**	**lowest**
External shell ions	**9.8**	**8.6**	**3.4**	**2.0**
Internal shell ions	4.6	4.2	8.0	9.4
Deeply buried ions	0.1	1.6	2.7	3.3
Duplex neutralization	91%	90%	88%	92%

The number of ions in the *external* shell clearly emerges as the key ‘order parameter’ that correlates well with the propensity of the four duplexes to condense. In other words, the greater the number of ions in the *external* shell of the duplex, the more readily the duplex condenses.

**Figure 5.**
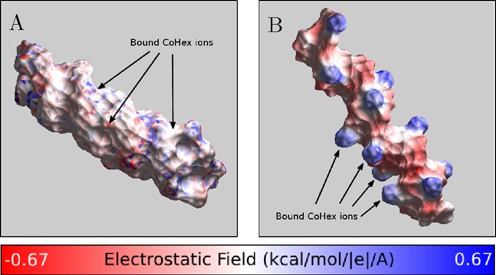
Charge neutralization patterns of NA duplexes by bound CoHex ions, assessed by the strength of the electric field near the NA-CoHex complex surface. (**A**) A-form mixed sequence RNA with CoHex counterions, which bind mostly in the major groove. (**B**) B-form mixed sequence DNA with CoHex ions, which are bound mostly externally. The specific snapshots are chosen to illustrate the *internal* (A) and *external* (B) binding modes from Figure 3 and reflect the actual average binding preferences; each snapshot has 15 bound (near neutralizing) CoHex ions, and is taken from the corresponding 320 ns-long all-atom MD simulation described in ‘Materials and Methods’. See Supplementary Data for a detailed visual characterization of CoHex ion distributions around these structures. The field is computed 3 Å away from the NA-CoHex complex molecular surface.

To explain the unique role that the distribution of bound CoHex ions relative to the helical axis plays in determining the major differences in condensation propensity between A- and B-form duplexes, we compare the charge neutralization patterns of mixed sequence B-form DNA and A-form RNA helices, Figure 5. To characterize these patterns we calculate the electric field at the external surface of NA-CoHex complexes (the connection between distribution of electric field strength in space and the system's electrostatic energy (∝ ε (∇ϕ)^2^) (68) motivates this type of analysis). The different preferable CoHex binding modes for B- and A-form duplexes create distinctive patterns of the net electric field at the duplex surface.

At ∼90% neutralization of the duplex charge, which is a precursor for NA–NA association (11,69), there is a substantial difference in the field patterns around B-DNA and A-RNA, Figure 5. The strong, localized field of the mobile and correlated 3+ counterions bound mainly at the surface of the phosphate backbone of the B-form duplex is not compensated locally by the more uniform background field of the duplex, thus resulting in a strong alternating pattern of electrostatic field along the surface of the phosphate backbones of the B-DNA, Figure 5B. This mobile alternating pattern makes it possible, by an appropriate mutual arrangement (21) of the duplexes in the NA–NA complex, to decrease the electrostatic energy substantially and thus create the NA–NA attraction necessary for the condensation (14). In contrast, in the A-form geometry, found in RNA and the hybrid structure, CoHex ions bind mostly internally, in the major groove, where the potential is initially much stronger than at the outside surface. The strong, highly correlated CoHex binding leads to a more uniform suppression of the electric field and its smaller spatial variation around the RNA-CoHex complex (Figure 5A). The net result for the A-form structures are nearly uniformly charged duplexes: bringing together uniformly charged cylinders cannot lower the electrostatic energy of the system, which would be necessary to generate an effective attraction. Implicit in the above reasoning is the assumption that no drastic re-distribution of CoHex ions bound to individual duplexes occurs when the duplexes approach each other at distances relevant for condensation. This approximation is borne out by single molecule DNA condensation experiments at mM CoHex concentrations (70), and by our simulations, see Supplementary Data.

We can now rationalize the more subtle structure-condensation relationships within B-family helices (mixed sequence versus homopolymeric DNA) and A-like form helices (RNA versus DNA:RNA hybrid), Table 1. Changing numbers of *external* shell ions can explain differences in condensation of the homopolymeric B′-DNA and the canonical B-form mixed sequence DNA. To trace the origin of the change, we performed another MD simulation of the homopolymer with the structure restrained in the canonical B-form conformation rather than the B′-form observed via CD and used in the simulations. This additional simulation shows the same 91% duplex neutralization by all bound CoHex ions with approximately the same number of ions bound in the *external* shell (9.4 as compared to 9.8 ions in B′-form). Similarly, no CoHex ions are found closer than 7 Å to the helical axis, compared to 1.6 ions bound to GpC steps in the mixed sequence DNA duplex.

Thus, it is not the minor structural difference between B′ and canonical B-form that accounts for the observed differences in condensation. Instead, specific sequence details; e.g. the presence of GpC steps, are responsible for the smaller number of bound CoHex ions at the external DNA surface. The GpC steps, which serve as additional binding sites in mixed sequence DNA (65), bind about two CoHex ions (see Table 1) reducing CoHex binding to the phosphate groups in mostly the *external* shell which is key to condensation.

To address the differences between the pure RNA and the DNA:RNA hybrid A-like form structures, we analyzed the duplex configurations from the MD trajectories and found very similar distributions of the phosphorus–phosphorus distances across the major groove, but a wider spread of the phosphate group oxygen orientations in the DNA strand of the DNA:RNA hybrid compared to the RNA strands (not shown). The less restrictive sugar-phosphate backbone of the DNA strand (56) allows orientations of the phosphate groups where unbridging oxygens are more directed toward the outside of the duplex. We suggest that such orientations are less favorable for CoHex binding inside the major groove of the hybrid, and promote the binding in the *external* shell, increasing the number of CoHex ions at the hybrid duplex external surface.

Thus, two A-like structures, having practically the same helical geometry but different distributions of the phosphate group orientations, can have different bound counterion distributions with respect to the helical axis, resulting in different condensation propensities.

## DISCUSSION

Our analysis suggests that the spatial distribution of ions around a NA double-helix has a dramatic effect on its ability to interact with neighboring helices. Specifically, the distribution of bound CoHex ions relative to the helical axis is the key ‘order parameter’: at near neutralizing conditions necessary for condensation it is the number of externally bound ions, rather than net bound, that predicts the experimentally observed condensation propensities of the short NA duplexes. We have rationalized the connection between location of the bound ions and condensation propensity by general electrostatic arguments that explain why DNA condenses more readily than RNA.

**Figure 6.**
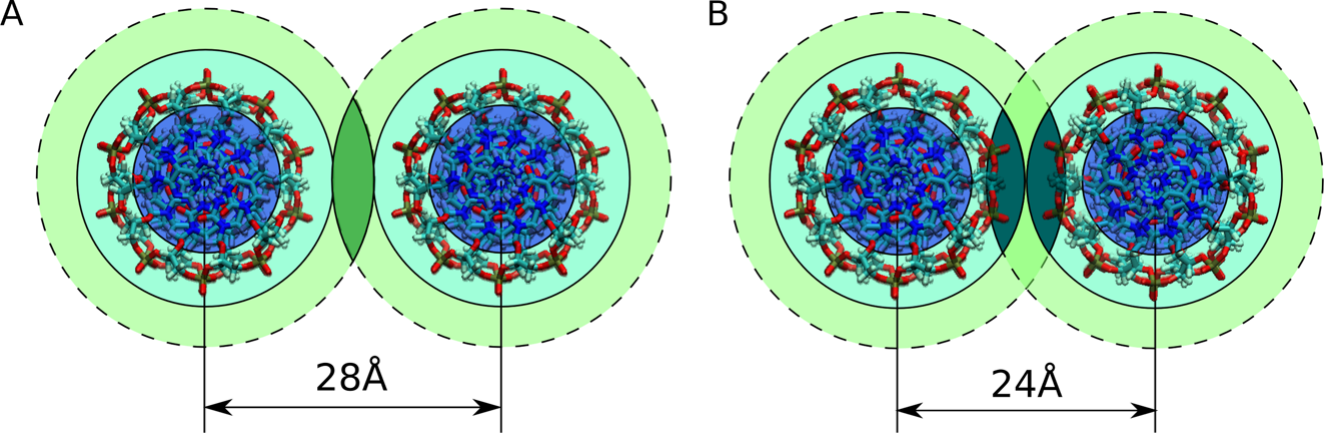
Schematic of (A) the *external–external* and (B) *external–internal* CoHex shell overlaps at different interaxial distances. The shell colors correspond to Figure 3. The overlapping shell regions are indicated by a darker color to guide the eye to the differences between (A) and (B).

The following argument further supports the connection between the counterion distribution around NA and condensation induced by multivalent counterions. Consider two parallel NA duplexes, with externally bound CoHex ions, approaching each other. An ion bound to duplex 1 experiences attractive net force from the nearly neutralized opposite duplex 2; this force becomes substantial when the ion on duplex 1 finds itself in one of the binding shells of duplex 2, i.e. when the shells overlap. This increase of the ion attraction in the shell overlapping region implies both non-specific binding of the ions to the NA surface and a correlation between the bound ions. The CoHex binding shells, Figure 3, for adjacent parallel duplexes can overlap in two different patterns, as depicted in Figure 6: *external–external* and *external–internal*. In the former, the axial separation is about 28 Å, while the overlap of *external* and *internal* shells results in smaller interaxial distances, 24–26 Å; the specific pattern is determined by the axial separation. Due to steric restrictions, overlap of the *internal–internal* shells is insignificant and can be ignored.

Remarkably, the experimentally reported interaxial distances between CoHex condensed DNA molecules are 27–28 Å (12,71). At these distances, the only significantly overlapping shells are the *external* ones, providing additional support for our proposal linking externally bound ions to condensation.

Finally, we comment on experimental observation that RNA eventually begins to condense at higher CoHex concentrations (30). While the precise spatial arrangement of RNA duplexes in CoHex-induced aggregates is unknown, it is difficult to imagine the absence of close side-by-side helix packing in such aggregates. If RNA packing is similar to that in DNA aggregates, we predict that RNA condensation should occur at interhelical distances smaller than those found in DNA aggregates. This is because at 27–28 Å interaxial distance, only the nearly empty RNA *external* shells overlap, while at shorter distances the attractive contribution can originate from counterions in the populated *external–internal* shell overlap, Figure 6B. Such a decrease in the duplex–duplex separation is likely accompanied by both the entropy loss and appreciable increase in the residual electrostatic repulsion. Note that end-to-end stacking of the RNA duplexes in the presence of CoHex was observed in the X-ray scattering experiments (30). This end-to-end RNA duplex interaction may result from favorable base-stacking interactions between charge compensated molecules (31) and may potentially be promoted by the solvent exposed bound CoHex ions at the ends of the RNA duplexes seen in our simulations, see Supplementary Data.

## CONCLUSION

This work combines experiment and atomistic modeling to propose a mechanistic picture of counterion-induced condensation of NA and resolves differences in their condensation propensities. The picture explains an unexpected experimental finding that NA condensation induced by trivalent counterion CoHex varies markedly between various short 25 bp double-helical structures, from RNA which largely resists condensation, to a homopolymer DNA which is most susceptible to it. A unique feature of our proposed mechanism is the relationship between NA duplex condensation and the location of preferential counterion binding relative to the NA helical axis. Specifically, condensation propensity is determined by the fraction of counterions bound to the external (outermost) surface of the double-helix. We found a significant difference, up to 5-fold, in the fraction of CoHex ions bound to the external surfaces of our different NA constructs. For example, in the simulated poly(dA):poly(dT) DNA structure 68% of the bound CoHex ions bind externally and can contribute to the NA–NA attraction and hence condensation, while in the mixed sequence dsRNA of the same size, most CoHex ions (87%) are bound internally (inside the major groove), which explains the observed resistance to condensation. In contrast to many previous studies, we make a clear distinction between the total number of counterions bound to NA and the fraction of counterions bound to the outermost surface. The overall strength of counterion-NA binding does not solely determine the condensation propensity; instead, the specific structural feature of bound ion distributions is of the most important influence. Multivalent ion binding to RNA is stronger than to DNA, yet the former resists condensation. Importantly, it is not simply variation in NA helical structure parameters that ultimately affects the condensation propensity: we find that duplexes with the same helical parameters can also have different condensation propensities.

It is beyond the scope of the current study to model condensed DNA states using MD simulations and to suggest specific modes of NA–NA association (e.g. ‘bridge’ versus ‘zipper’ etc.). Nevertheless, it is useful to examine our findings in the light of previous theoretical studies that attempt to explain the differences in DNA and RNA condensation (32) based on the differences in the helical structure. The assumptions (32) made about the preferential placement of bound CoHex ions and the purely geometric interpretation are inconsistent with our experimental data and the results of atomistic simulations of CoHex binding. Our results show that in DNA the majority of bound CoHex are outside of the major groove and that the peaks of CoHex distributions overlap at the NA–NA distances that correspond to the condensed phase. While NA condensation depends on a complex interplay between various structural and sequence features, our coupled experimental and theoretical results suggest a new model in which a single parameter—the fraction of externally bound multivalent counterions—connects the NA condensation propensity with geometry and sequence dependence of CoHex binding.

## SUPPLEMENTARY DATA

 Supplementary Data are available at NAR Online.

SUPPLEMENTARY DATA
