# Supplementary material for: Why double-stranded RNA resists condensation
Source: Nucleic Acids Res. 2014 Aug 14;42(16):10823–31. doi: 10.1093/nar/gku756 (PMC4176364; doi:10.1093/nar/gku756)
Supplement: SUPPLEMENTARY DATA [file supp_42_16_10823__index.html]

Why double-stranded RNA resists condensation — Why double-stranded RNA resists condensation — Why double-stranded RNA resists condensation — Why double-stranded RNA resists condensation — SUPPLEMENTARY DATA 

# Why double-stranded RNA resists condensation

## SUPPLEMENTARY DATA

**Files in this Data Supplement:**

- SUPPLEMENTARY DATA
