# Supplementary material for: Why double-stranded RNA resists condensation
Source: Nucleic Acids Res. 2014 Aug 14;42(16):10823–31. doi: 10.1093/nar/gku756 (PMC4176364; doi:10.1093/nar/gku756)
Supplement: SUPPLEMENTARY DATA [file supp_gku756_nar-01307-f-2014-File002.pdf]

Published online *Nucleic Acids Research, 2014, Vol. xx, No. xx* **1–4**
doi:10.1093/nar/gkn000

SUPPLEMENTARY DATA to: Why double-stranded RNA resists condensation

Igor S. Tolokh [1,‖], Suzette A. Pabit [2,‖], Andrea M. Katz [2], Yujie Chen [2], Aleksander Drozdetski [3], Nathan Baker [4], Lois Pollack [2,*] and Alexey V. Onufriev [1,3, *]

[1]Department of Computer Science, Virginia Tech, Blacksburg, VA 24061, USA, [2]Cornell University, School of Applied and Engineering Physics, Ithaca, NY 14853-3501, [3]Department of Physics, Virginia Tech, Blacksburg, VA 24061, USA, and [4]Applied Statistics and Computational Modeling Group, Pacific Northwest National Laboratory, Richland, WA 99352, USA

Details of CoHex ion distribution around DNA and RNA duplexes

In addition to the radial distributions of the bound CoHex ions around NA molecules, shown in Figure 3 of the main text, we present here the spatial distributions of CoHex around the DNA and RNA duplexes.

In the DNA, Figure S1, most of CoHex ions bound to phosphate groups are in the external ion binding shell (12 – 16 Å from the helical axis) "hovering over" the phosphate backbone and the minor groove. Only a small fraction ($\sim$ 1/3) of the bound ions are in the major groove with almost all of them being in the internal ion binding shell (7 – 12 Å from the helical axis). CoHex density is substantially reduced near the terminal phosphate groups of each DNA strand.

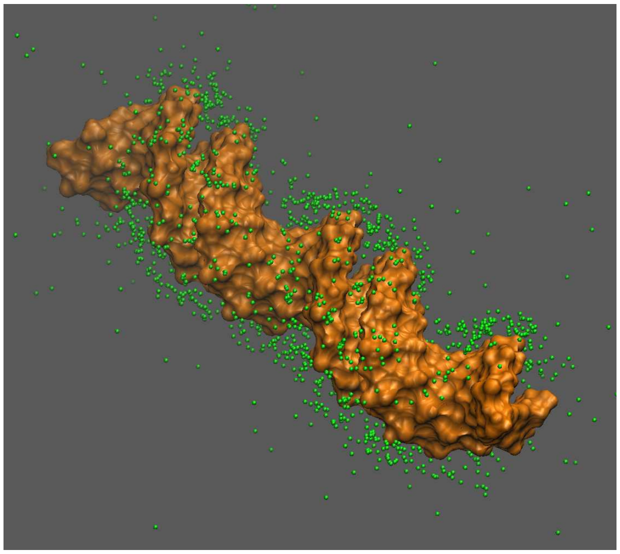

Figure S1. Spatial distribution of CoHex ions around homopolymeric 25 bp poly(dA):poly(dT) DNA. Green dots represent positions of CoHex ions per nanosecond sampled over a 100-ns region of the MD trajectory.

In the RNA, Figure S2, CoHex ions bind almost exclusively inside the major groove. Only a tiny fraction of ions bind to the surface of the RNA minor groove or to the external surface of the sugar-phosphate backbone. Note the exposed bound CoHex ions at both ends of the duplex where the major grooves end. These ions may participate in end-to-end attraction of the RNA duplexes that may result in creation of longer filaments.

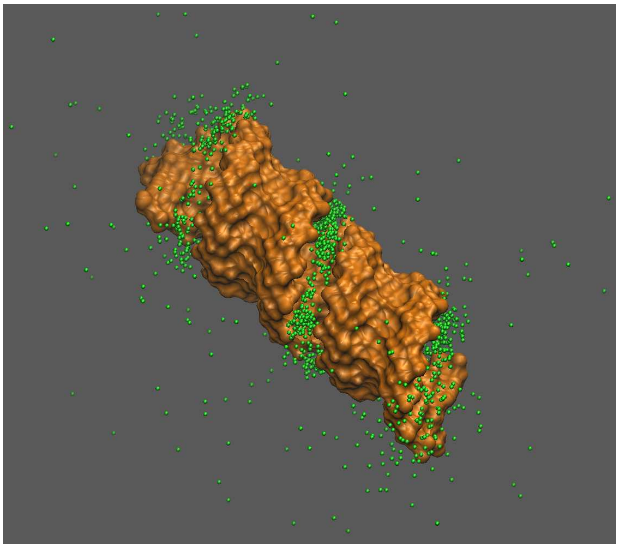

Figure S2. Spatial distribution of CoHex ions around 25 bp mixed sequence RNA. Green dots represent positions of CoHex ions per nanosecond sampled over a 100-ns region of the MD trajectory. Most of CoHex ions are bound inside the major groove of RNA within 12 Å from the helix axis.

Sequence specificity has some effect on the distribution of the bound CoHex ions. For example, the mutual configuration of Guanine base oxygens in the GpC steps leads to a relatively high, localized CoHex affinity in the major groove, Figure S3.

*To whom correspondence should be addressed. Email: alexey@cs.vt.edu or lp26@cornell.edu. ‖First co-authors who contributed equally.

© 2014 The Author(s)
This is an Open Access article distributed under the terms of the Creative Commons Attribution Non-Commercial License (http://creativecommons.org/licenses/by-nc/2.0/uk/) which permits unrestricted non-commercial use, distribution, and reproduction in any medium, provided the original work is properly cited.

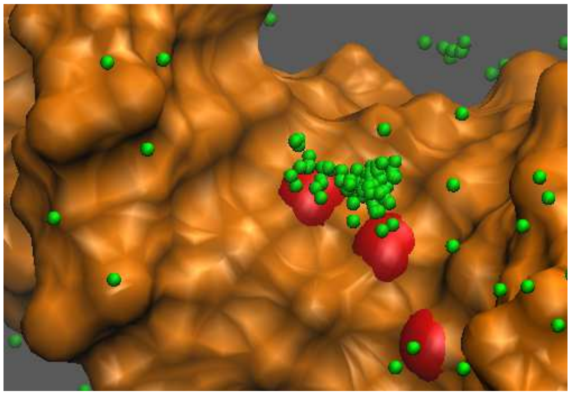

Figure S3. Deeply buried CoHex ion bound to a GpC step in the mixed sequence DNA. Zoomed-in view of the major groove. Green dots represent positions of the ion sampled every 1 ns over 100 ns part of the MD trajectory. Red spheres represent Guanine base oxygens.

Insignificant re-distribution of bound CoHex ions between the bound ion shells around NA upon formation of a pair of interacting duplexes

The discussion in the main text assumed that no substantial re-distribution of bound CoHex between external and internal ion shells around a single NA molecule occurs when the two duplexes approach each other. Here we verify that an approaching RNA duplex does not “pull” a substantial number of CoHex ions from the internal shell of the other duplex.

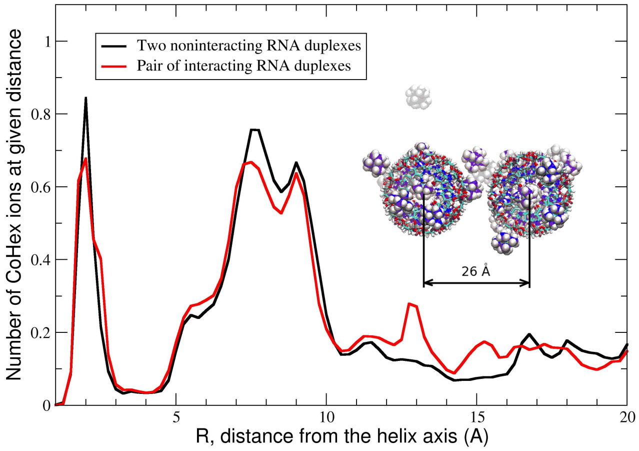

Figure S4. Combined CoHex distribution of two independent 25 bp RNA duplexes (black curve) vs. the distribution in a pair of interacting duplexes (red curve) at 26 Å separation. The combined distribution is calculated as a sum of two individual distributions shifted by 26 Å.

For RNA, this verification is presented in Figure S4, where we have compared the sum of independent simulated CoHex distributions around single RNA duplex with the CoHex distribution around a pair of such duplexes. Without loss of generality, the RNA molecules were restrained to canonical A-form. In the case of the pair of duplexes, the molecules were kept at a 26 Å distance between their helical axes. This separation allows the tails of CoHex distribution formed by the externally bound CoHex ions to overlap. As seen from Figure S4, the presence of the second RNA duplex has an insignificant effect on the distribution of strongly bound CoHex within 12 Å from the axis of the first duplex. In fact, the small differences between two curves in Figure S4 average out almost completely upon integration that yields the total numbers of bound ions: 13.5 ions in both cases. The difference in the ion densities beyond 12 Å is due to a small re-distribution of CoHex in the external shells of two interacting duplexes.

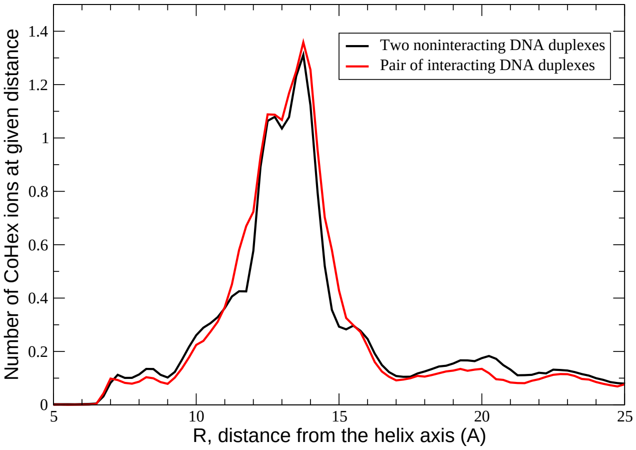

Figure S5. Combined CoHex distribution of two independent 25 bp poly(dA):poly(dT) DNA duplexes (black curve) vs. the distribution in a pair of interacting duplexes (red curve) at 26 Å separation. The combined distribution is calculated as a sum of two individual distributions shifted by 26 Å.

Figure S5 presents evidence for the absence of substantial re-distribution of bound CoHex between external and internal ion shells around DNA duplexes when they approach each other. The sum of independent simulated CoHex distributions around a single DNA (poly(dA):poly(dT)) duplex is compared with the CoHex distribution around a pair of such duplexes. As in the case of RNA, two DNA molecules were kept at a 26 Å distance between their helical axes. The numbers of CoHex ions in the internal shell of a single duplex differs from the corresponding number in the case of a pair of interacting duplexes by less than 0.2 ions. A somewhat greater difference, 1.1 ions, is found between the external shells of the duplexes, in the case of the interacting duplex pair compared to the case of non-interacting DNA duplexes. Since the number of all bound CoHex ions around the DNA pair differs insignificantly from the corresponding number of ions around the two non-interacting DNA duplexes, the difference of 1.1 ions is suggested to be due to a re-distribution of the bound CoHex ions only within the external shells of the interacting DNA duplexes. We suggest that such a re-distribution will be substantially reduced in the case of hexagonally packed DNA aggregates when the six external shell overlapping regions are formed around each DNA duplex.

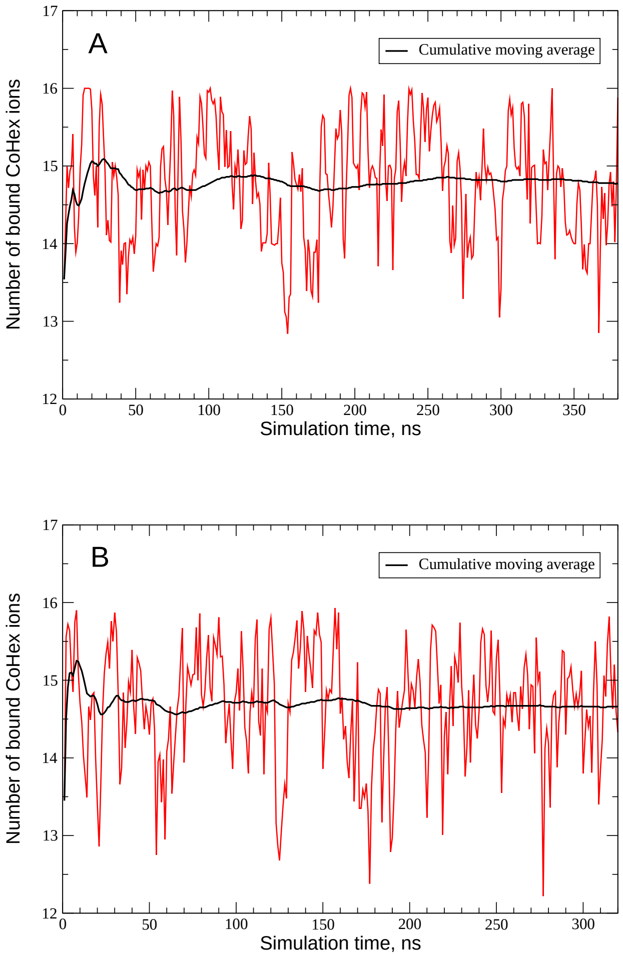

Figure S6. Number of bound CoHex ions within 16 Å from the duplex helical axis as a function of simulation time. (A) RNA and (B) poly(dA):poly(dT) DNA. Each point is a time average over 1 ns, cumulative moving averages are shown as well.

Hundreds of nanoseconds of all-atom MD are needed to adequately represent the fluctuating CoHex atmosphere around RNA duplex

The number of bound ions is expected to fluctuate significantly around NA duplexes. These fluctuations affect the accuracy of the calculated averages of the number of bound CoHex ions presented in the main text. As seen from Figure S6, a $\sim$300 ns averaging window is sufficient to obtain well converged averages. In calculations of CoHex distributions and average numbers of bound CoHex ions in different ion binding shells around the NA duplexes we have ignored the first 40 ns of each MD trajectory to allow for equilibration of the ionic atmosphere.

CoHex ion distribution around NA is robust to the choice of water model used in MD simulation

Here we investigate possible dependence of the CoHex ion distribution around a short NA duplex on the choice of the water model used in MD simulations. We have carried out a simulation of the CoHex atmoshere around the homopolymeric 25 bp poly(dA):poly(dT) DNA duplex using the TIP4P-Ew (1) water model instead of the TIP3P (2) model employed in the simulations reported in the main text; all other simulation parameters remained the same. The resulting distribution of CoHex counterions is shown in Figure S7, where it is compared with the original (main text) CoHex distribution obtained using TIP3P water model. The conclusion is that the CoHex distribution is robust to the choice of water model.

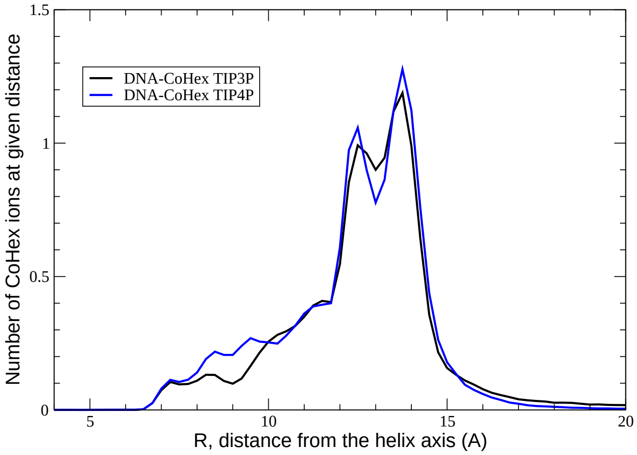

Figure S7. Robustness of the computed CoHex distribution around NA to the choice of the water model. Shown are CoHex distributions around 25 bp poly(dA):poly(dT) DNA duplex simulated in TIP3P (discussed in the main text) and TIP4P water models.

Structural changes in the RNA duplex caused by CoHex binding have relatively small effect on the electrostatic potential around the duplex

As discussed in the main text, the binding of CoHex causes conformational changes in the A-form structures of RNA and DNA:RNA hybrid. To investigate the possible effect of these changes on the electrostatic potential around the RNA duplex, we have repeated the calculation shown in Fig. 4 of the main text, but now for the RNA structure with the CoHex-induced conformational changes, Figure S8. As in the case of RNA before CoHex binding, Figure 4 of the main text, the strongest potential occurs in the major groove (the maximum value is +21.29 kcal/mol/$|e|$). Just like in the case of canonical RNA structure, the positive electrostatic potential in the internal binding shell (major groove) is at least 10 kcal/mol/$|e|$ stronger than in the external shell. Therefore, the rationale given in the main text for the preferential internal binding of CoHex in A-like duplexes is robust to CoHex induced conformational changes in the RNA. Given the high similarity between RNA and DNA:RNA hybrid structures in CoHex, we expect the conclusion to hold for the hybrid as well.

REFERENCES

1. Hans W. Horn, William C. Swope, Jed W. Pitera, Jeffry D. Madura, Thomas J. Dick, Greg L. Hura, and Teresa Head-Gordon. Development of

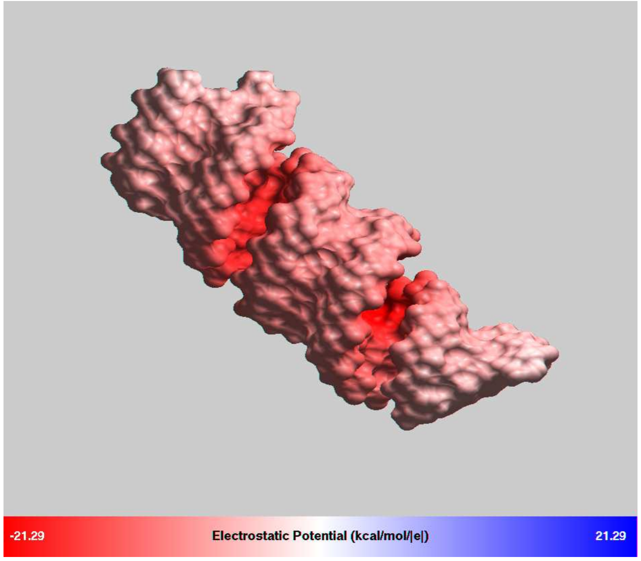

Figure S8. Electrostatic potential at the surface of 25 bp RNA duplex after equilibration with CoHex ions in solution. As in Figure 4 of the main text, CoHex ions were removed prior to calculating the potential. The graphics is by GEM package (3).

an improved four-site water model for biomolecular simulations: TIP4P-Ew. *The Journal of Chemical Physics*, 120(20):9665–9678, May 2004.

2. William L. Jorgensen, Jayaraman Chandrasekhar, Jeffry D. Madura, Roger W. Impey, and Michael L. Klein. Comparison of simple potential functions for simulating liquid water. *The Journal of Chemical Physics*, 79(2):926–935, 1983.
3. John C Gordon, Andrew T Fenley, and Alexey Onufriev. An analytical approach to computing biomolecular electrostatic potential. II. Validation and applications. *The Journal of Chemical Physics*, 129:075102, 2008.
